# Absence of the Electric Aharonov-Bohm Effect due to Induced Charges

**DOI:** 10.1038/srep14279

**Published:** 2015-09-22

**Authors:** Rui-Feng Wang

**Affiliations:** 1 Department of Physics, Beijing Jiaotong University, Beijing, 100044, China

## Abstract

This paper states that the induced charge should not be neglected in the electric Aharonov-Bohm (A-B) effect. If the induced charge is taken into account, the interference pattern of the moving charge will not change with the potential difference between the two metal tubes. It means that the scalar potential itself can not affect the phase of the moving charge, and the true factor affecting the phase of the moving charge is the energy of the system including the moving charge and the induced charge.

In classical physics, the concept of “force” is the most important, all the phenomena can be explained by the forces acting on the objects. In classical electrodynamics, the Lorentz force acting on a charge is determined by the electric field **E** and the magnetic field **B** at the position of the charge. So, the electric field **E** and the magnetic field **B** are considered as more fundamental quantities than the scalar potential *φ* and the vector potential **A**. But, in quantum mechanics, what appear in the Schrödinger equation are the scalar potential *φ* and the vector potential **A** instead of **E** and **B**. So, some physicists asserted that the potential functions *φ* and **A** are more fundamental than **E** and **B**^1^. Just for this reason, Y. Aharonov and D. Bohm predicted a new effect named by their names later^2^. This new effect asserts that the phase of a moving charge will be changed by the potential functions *φ* and **A**, even though the charge always move in a region where both **E** and **B** are zero, but the *φ* and **A** are not zero. This effect includes the electric A-B effect and the magnetic A-B effect. The magnetic A-B effect has been studied extensively in both theory and experiments^3^,^4^,^5^,^6^,^7^,^8^,^9^,^10^,^11^,^12^. The existence of the magnetic A-B effect has been supported by some experiments^3^,^5^. The theoretical and experimental studies on the magnetic A-B effect before 1989 have been well reviewed in the Ref. 8. But the electric A-B effect was much less studied^13^,^14^,^15^,^16^,^17^,^18^. Some experiments^13^,^14^,^15^,^16^ attempted to observe the electric A-B effect, but none of them could completely avoid the classical force acting on the moving charge due to the magnetic or electric fields in the experiment. The quantitative experimental result about the influence of scale potential has not been reported. Recently, a new experimental method^18^ has been advised, in which the moving electron is replaced by the moving hydrogen ion in order to lower the speed of the moving charge.

This paper will focus on the electric A-B effect in theory, especially, the possible experiment with two metal tubes proposed in the seminal paper by Aharonov and Bohm. It is found that a very important factor was neglected in their paper^2^, which is the induced charge on the inner surfaces of the metal tubes. If this induced charge is taken into account, we will find that the phase of the moving charge will not change with the electric potential difference between two metal tubes. So, it is more convincing to state that the real factor affecting the phase of a moving charge is the energy of the system including the moving charge and the induced charge, but not the scalar potential itself. The similar conclusions about the magnetic A-B effect^6^,^10^,^11^,^12^ and its theoretical proof^10^ have also been proposed before.

## The electric A-B effect with induced charges neglected

**Figure 1:**
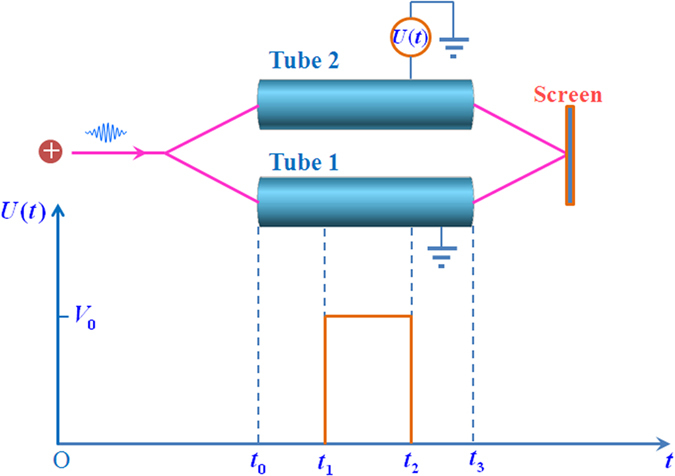
Schematic experiment to demonstrate the electric A-B effect. *U*(*t*) represents the external voltage generator. The charge enters into the tubes at *t* = *t*_0_, the external voltage generator is switched on at *t* = *t*_1_, the external voltage generator is switched off at *t* = *t*_2_, then, the charge leave the tubes at *t* = *t*_3_.

First, let us repeat the possible experiment proposed by Aharonov and Bohm to demonstrate the electric A-B effect. As depictured in Fig. 1, a coherent charge beam is split into two parts and each part enters into a separate long cylindrical metal tube. After the beams pass through the metal tubes, they are combined to interfere coherently at the screen. By means of time-determining electrical “shutters” the charge beam is divided into wave packets, the length of each wave packet is long compared with its wavelength but short compared with the length of the metal tube. To analysis this experiment in more detail, we suppose the moving charge enter into the metal tubes at *t*_0_, comes out from the tubes at *t*_3_, in addition, *t*_0_ < *t*_1_ < *t*_2_ < *t*_3_. During the time interval from *t*_1_ to *t*_2_, while the moving electron is well inside the tubes, an electric potential difference *V*_0_ is applied between these two tubes. For example, the tube 1 is always connected to the zero potential point, and the tube 2 is connected to an external voltage generator, which makes the electric potential of the tube 2 to alternate in time as following:


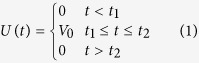


To keep the potentials of the two tubes being zero in *t* < *t*_1_ and *t* > *t*_2_, the metal tube 2 should also be connected with the zero potential point in these two time intervals. Otherwise, the collision of the ions and the charges accumulation will make the potential of the tube 2 uncontrollable.

Let’s discuss this problem in the following situations:

### The first situation

The external voltage generator is switched off, 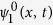 and 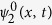 represent the wave functions of the parts passing through the tubes 1 and 2, respectively, which are unperturbed by the external electric potential; 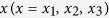 is the coordinate of the moving charge. The total wave function 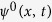 is:


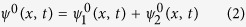



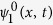
 and 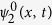 are determined by the following equations:


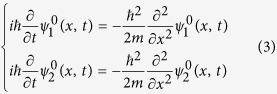


where 
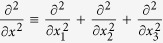
.

### The second situation

The external voltage generator is switched on. 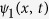 and 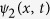 represent the wave functions perturbed by the external electric potential. The total wave function 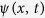 is:


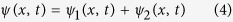



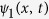
 and 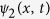 are determined by the following equations:


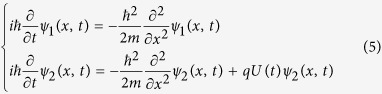


Then, comparing the eq. (3) and (5), the wave functions 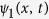 and 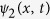 for the charge in the two beams are given by:


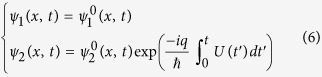


When *t* > *t*_2_, the total wave function become:


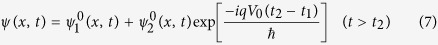


Comparing the equations (2) and (7), after the two beams come out from the tubes, an additional phase difference between these two beams appears in the second situation, which is


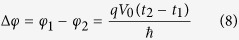


So, when these two beams meet at the screen, the interference pattern will change with the value of 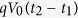.

The discussion above is quite similar to the original paper by Aharonov and Bohm^2^, which also appears in some modern quantum mechanics textbooks^19^. But, in the discussion above, no induced charge was taken into account. No induced charge appearing on the surfaces of the metal tubes means that the electric field of the moving charge exists in all the space. So, in these two situations above, the electric field of the moving charge is not zero in the interior of the metal, furthermore, this electric field can penetrate the metal tube and exists in the region outside the metal tubes. Obviously, the discussion above is not reliable in principle. The induced charges should be taken into account.

In classical electrodynamics, if a charge is placed outside a metal surface, the induced charges will appear on the metal surface, where the metal surface is idealized as a mathematical surface of zero thickness. In quantum mechanics, Ref. 20 showed that the induced charges appear in a 0.2 *nm* thick layer near the metal surface in reality, but, the potential energy between the external charge and the induced charges can be described to a good approximation by the results of classical electrodynamics.

**Figure 2:**
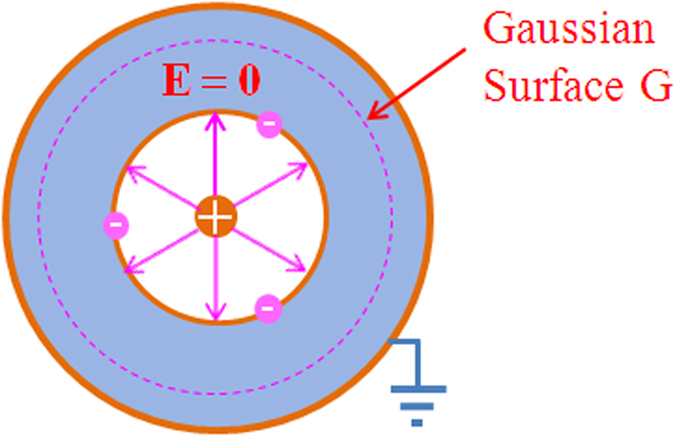
A charge is placed in a metal cavity, the induced charges will appear on the inner surface of metal cavity ensuring that the electric field E at every point within the metal is zero.

As depicted in the Fig. 2 a charge *q* is placed in a cavity that is totally within the metal, and the outer surface of the metal is connected to the zero potential point. Suppose the total induced charges on the inner surface is 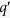. As long as there are free electrons in the metal, the electric field **E** at every point within the metal should be zero. As long as the Coulomb’s law holds, or in other words, the potential of a point charge *q* is 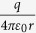, the Gauss’s law should hold too. So, if we draw a Gaussian surface G surrounding the cavity, for **E** = 0 everywhere on the Gaussian surface:


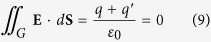


Then, we have


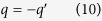


### The electric A-B effect with influence of the induced charges

So, in experiments, once the moving charge *q* (suppose *q* = ±*e*) get into the metal tubes, an induced charge 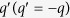 will appear on the inner surfaces of the metal tubes. (If the moving charge *q* is a proton, the induced charge 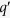 will be an electron; If the moving charge *q* is an electron, then the induced charge 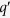 will be a hole in the Fermi gas of the metal tube. The induced charge 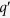 is located on the inner surfaces of the tubes.) At the same time, another induced charge 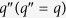 will appear on the outer surfaces of the metal tubes. During the time interval from *t*_0_ to *t*_1_, both the tubes are connected to the zero potential point, so, the induced charge 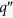 on the outer surfaces of the metal tubes will flow into the zero potential point. Therefore, only the induced charge 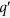 on the inner surfaces of the tubes needs to be taken into account.

When the charge *q* moves in the metal tubes, for the induced charge 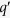 appears on the inner surfaces of the tubes, the electric field of the moving charge *q* will be shielded by the induced charge 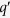, and the resultant electric field produced by the charges *q* and 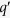 only exists in the region enclosed by the inner surface of the metal tubes. For the moving charge *q* and the induced charge 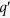 attract each other, the coordinate *y*(*y* = *y*_1_, *y*_2_, *y*_3_) of the induced charge 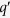 is dependent on the coordinate *x*(*x* = *x*_1_, *x*_2_, *x*_3_) of the moving charge *q*. Therefore, the wave function of the induced charge 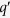 changes with the position of the moving charge *q*, at the same time, the wave function of the moving charge *q* is also perturbed by the induced charge 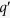
21,22. So, both the moving charge *q* and the induced charge 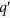 are not free particles, we should take the moving charge *q* and the induced charge 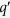 as a system. Let 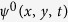 and 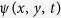 represent the wave functions of this system with the external voltage generator being switched off or on, respectively.

In the region outside the metal tube (*i.e. t* < *t*_0_ or *t* > *t*_3_), the moving charge is a free particle and has no relationship with the induced charges and the potential difference *U*(*t*) between the two tubes. So, we need only to discuss the revolution of the wave function of the charges *q* and 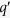 in the region enclosed by the metal tubes, *i.e*. the time dependence of the wave function from *t*_0_ to *t*_3_.

So, ***the third situation***: the external voltage generator is switched off, but, the induced charge 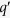 is included. Then the total wave function is:


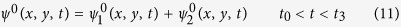


where 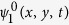 and 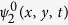 represent the wave functions of the parts passing through the tubes 1 and 2, respectively, which are unperturbed by the external electric potential *U*(*t*). The wave functions 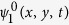 and 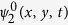 satisfy the following Schrödinger equation:


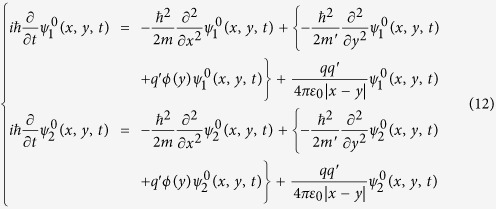


where, *t*_0_ < *t* < *t*_3_; 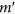 is the mass of *t*he induced charge 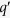; ϕ(*y*) is the potential function experienced by the induced charge 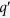 in the metal tubes, which ensures that the induced charge 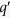 can only move in the interior of the metal and cannot leave out from the surfaces of the metal tubes, (noticing: ϕ(*y*) is independent of the potential difference *U*(*t*) between the two me*t*al tubes.); 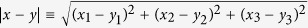; and 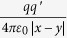 is the interaction energy between the moving charge *q* and the induced charge 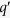.

***The fourth situation:*** the external voltage generator is switched on, and the perturbed wave function 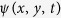 of the system will become


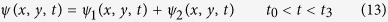


where 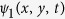 and 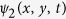 represent the wave functions perturbed by the external electric potential *U*(*t*). 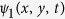 and 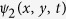 satisfy the following Schrödinger equations:


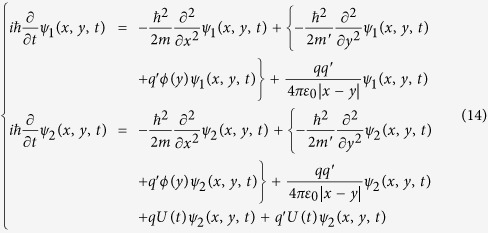


In the equations (14), *qU*(*t*) and 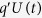 are the potential energies of the moving charge *q* and the induced charge 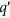 in the electric field *U*(*t*), respectively. For 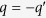, the sum of 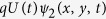 and 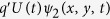 is zero *i.e*. the interaction energy between *q* and *U*(*t*) is completely counteracted by the interaction energy between 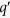 and *U*(*t*). Therefore, the equations (14) will become:


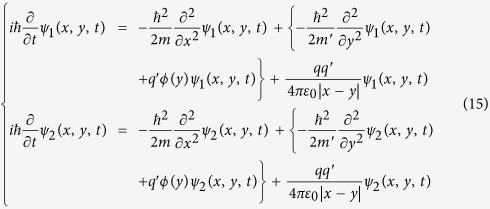


Now, the potential difference *U*(*t*) has disappeared from the eq. (15), *i.e*. the Schrödinger equations (15) are independent of the potential difference *U*(*t*), so, the wave functions 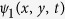 and 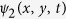 should also be independent of *U*(*t*). Comparing the equations (12) and (15), which are exactly same to each other, so, the following equations are obvious:


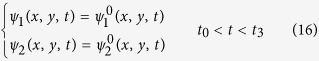


In this situation, the total wave function is:


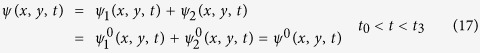


For 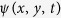 is the wave function of the charges *q* and 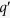 with the external potential difference being *U*(*t*), but, 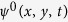 is the wave function with the external potential difference being zero, 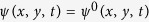 means that the wave function will not change with the potential difference *U*(*t*). So, when the two beams meet at the screen, the interference pattern will not change with *U*(*t*), *i.e*. the phase shift as eq. (8) predicted by the electric A-B effect will not appear.

## Discussions and Conclusions

Why does not the phase shift predicted by the electric A-B effect appear if the induced charge is included? Because, if there were no induced charge on the surfaces of the metal tubes, the electric field of the moving charge would exist in all the space, this field could penetrate the metal tubes and overlap with the electric field between the two tubes applied by the external voltage generator. While the moving charge *q* is in the tube 2, its potential energy is *qU*(*t*); while the moving charge *q* is in the tube 1, its potential energy is 0. According to quantum mechanics, the wave function of the moving charge is:


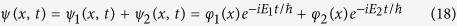


where *E*_1_ and *E*_2_ are the energies of the parts passing through the tube 1 and 2, respectively. They are given by:


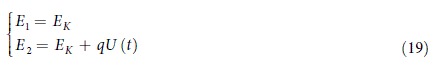


where *E*_*K*_ is the kinetic energy of the moving charge. Obviously 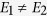, so, the time dependence of the 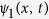 is different with that of 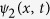. Therefore, when these two beams come out from the tubes, a phase shift due to the potential difference *U*(*t*) between the tubes will appear, this is the electric A-B effect predicted by Aharonov and Bohm. This analysis is consistent with the eq. (8). The eq. (8) shows the phase shift Δ*φ* is proportion to *qV*_0_, which represents the potential energy of the moving charge *q*. So, the eq. (8) strongly implies the phase shift Δ*φ* arise from the interaction energy between the moving charge *q* and the electric field *U*(*t*)^6^.

But in a real experiment, when the moving charge *q* moves in the metal tubes, there must be an induced charge 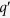 appearing on the inner surfaces of the metal tubes. Just for the appearance of the induced charge 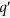, the resultant electric field produced by the moving charge *q* and the induced charge 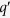 can only exist in the region enclosed by the inner surfaces of the metal tubes. So, this resultant electric field cannot overlap with the electric field between the tubes. The potential energy (related to *U*(*t*)) of the system including the moving charge *q* and the induced charge 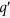 is zero no matter the moving charge *q* is in the tube 1 or tube 2. The wave function of the system:


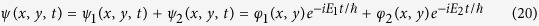


where, *E*_1_ is the energy of the system with the moving charge passing through the tube 1, *E*_2_ is the energy of the system with the moving charge passing through the tube 2. *E*_1_ and *E*_2_ are given by:


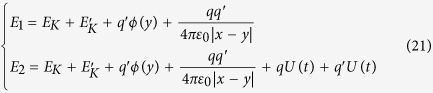


where, *E*_*K*_ and 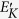 are the kinetic energies of the moving charge *q* and the induced charge 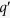; the other quantities are defined as above.

For 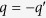, so:


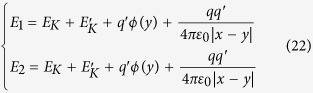


Obviously,


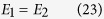


So, the time dependence of 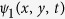 is same to that of 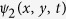. Therefore, when these two beams come out from the tubes, no phase shift due to the potential difference *U*(*t*) will appear. When these two parts meet at the screen, the interference pattern will not change with the potential difference *U*(*t*) between the two tubes.

In this situation, the potential difference *U*(*t*) between the two metal tubes still exists, but the phase shift due to *U*(*t*) does not appear. So, it is difficult to state the scalar potential can affect the phase of a moving charge; it is more reasonable to state that the real factor affecting the phase of a moving charge should be the potential energy of the system including the moving charge *q* and the induced charge *q*′.

## Additional Information

**How to cite this article**: Wang, R.-F. Absence of the Electric Aharonov-Bohm Effect due to Induced Charges. *Sci. Rep*. **5**, 14279; doi: 10.1038/srep14279 (2015).

## References

[b1] Aharonov Y. & Bohm D. Further considerations on electromagnetic potentials in the quantum theory. Phys. Rev. 123, 1511 (1961).

[b2] Aharonov Y. & Bohm D. Significance of electromagnetic potentials in the quantum theory. Phys. Rev. 115, 485 (1959).

[b3] Chambers R. G. Shift of an electron interference pattern by enclosed Magnetic flux. Phys. Rev. Lett. 5, 3 (1960).

[b4] Furry W. H. & Ramsey N. F. Significance of potentials in quantum theory. Phys. Rev. 118, 623 (1960).

[b5] Tonomura A. . Evidence for Aharonov-Bohm effect with magnetic field completely shielded from electron wave. Phys. Rev. Lett. 56, 792 (1986). 10033287 10.1103/PhysRevLett.56.792

[b6] Feĭnberg E. L. On the “special role” of the electromagnetic potentials in quantum mechanics. Sov. Phys. Usp 5, 753 (1963).

[b7] Erlichson H. Aharonov-Bohm effect—quantum effects on charged particles in field-free regions. Am. J. Phys. 38, 162 (1970).

[b8] Peshkin M. & Tonomura A. The Aharonov-Bohm effect (Springer- Verlag, Berlin, 1989).

[b9] Caprez A., Barwick B. & Batelaan H. Macroscopic test of the Aharonov- Bohm effect. Phys. Rev. Lett. 99, 210401 (2007). 18233196 10.1103/PhysRevLett.99.210401

[b10] Wang R. F. An experimental scheme to verify the dynamics of the Aharonov-Bohm effect. Chin. Phys. B 18, 3226 (2009).

[b11] Wang R. F. Interaction between a moving electron and magnetic flux in Aharonov-Bohm effect. arXiv:1312. 6253 (2013).

[b12] Wang R. F. A possible interplay between electron beams and magnetic fluxes in the Aharonov-Bohm effect. Front. Phys. 10, 100305 (2015).

[b13] Schmid H. in proc. 8 th European Congress on Electron Microscopy (eds Csanady A., Rohlich P. & Szabo D. ) 285–286 (Programme Committee of the 8 th Eur. Congr. On Electron Microsc, Budapest, 1984).

[b14] Washburn S., Schmid H., Kern D. & Webb R. A. Normal-metal Aharonov-Bohm effect in presence of a transverse electric field. Phys. Rev. Lett. 59, 1791 (1987). 10035332 10.1103/PhysRevLett.59.1791

[b15] Matteucci G. & Pozzi G. New diffraction experiment on the electrostatic Aharanov-Bohm effect. Phys. Rev. Lett. 54, 2469 (1985). 10031351 10.1103/PhysRevLett.54.2469

[b16] Van Oudenaarden A., Devoret M. H., Nazarov Yu. V. & Mooij J. E. Magneto-electric Aharonov-Bohm effect in metal rings. Nature 391, 768 (1998).

[b17] Weder R. The electric Aharonov-Bohm effect. J. Math. Phys. 52, 052109 (2011).

[b18] Schütz G., Rembold A., Pooch A., Prochel H. & Stibor A. Effective beam separation schemes for the measurement of the electric Aharonov-Bohm effect in an ion interferometer. arXiv: 1303.7140. 10.1016/j.ultramic.2015.06.016 26188995

[b19] Auletta G., Fortunato M. & Parisi G. Quantum Mechanics (CambridgeUniversity Press, 2009).

[b20] Lang N. D. & Kohn W. Theory of metal surfaces: induced surface charge and image potential. Phys. Rev. B. 7, 3541 (1973).

[b21] Barwick B., Gronniger G., Yuan L., Liou S. H. & Batelaan H. A measurement of electron-well interactions using transmission diffraction from nanofabricated gratings. J. Appl. Phys. 100, 074322 (2006).

[b22] Sonnentag P. & Hasselbach F. Decoherence of electron waves due to induced charges moving through a nearby resistive material. Braz. J. Phys. 35, 385 (2005).

